# Supplementary material for: Population-based cancer incidence and mortality rates and ratios among adults with intellectual disabilities in Scotland: a retrospective cohort study with record linkage
Source: BMJ Open. 2024 Aug 12;14(8):e084421. doi: 10.1136/bmjopen-2024-084421 (PMC11331995; doi:10.1136/bmjopen-2024-084421)
Supplement: online supplemental file 1 [file bmjopen-14-8-s001.pdf]

Supplementary Tables

S1. Cancer types categorised by ICD-10 codes from C00 – C97 Malignant Neoplasms.

ICD10 code category	ICD10 subcategory	Cancer area
C00-C14		Lip, oral cavity, & pharynx
C15-C26	 C15<br>C16<br>C22<br>C18-C21<br>C25	Digestive organs<br>Oesophageal<br>Stomach<br>Liver<br>Colorectal<br>Pancreas
C30-C39	 C33-34	Respiratory & Intrathoracic organs<br>Lung cancer
C40-C41		Bone & articular cartilage
C43-C44	C43<br>C44	Melanoma (skin)<br>Other malignant neoplasms (non-melanoma)
C45-C49		Mesothelial & soft tissue
C50		Breast
C51-C58	 C51<br>C53<br>C54-C55<br>C56	Female genital organs<br>Vulva<br>Cervical<br>Uterus<br>Ovary
C60-C63	 C61<br>C62	Male genital organs<br>Prostate<br>Testicular
C64-C68	 C64<br>C67	Urinary tract<br>Kidney<br>Bladder
C69-C72		Eye, brain & other parts of the CNS
C73-C75	 C73	Thyroid and other endocrine glands<br>Thyroid
C76-C80		Metastatic cancer of unknown primary origin (ill-defined, secondary, & unspecified sites)
C81-C96	 <br>C81 - C85<br>C91<br>C92<br>C96	Stated or presumed to be primary, of lymphoid, haematopoietic, and related tissue<br>Hodgkin's & Non-Hodgkin's lymphoma<br>Lymphoid leukaemia<br>Myeloid leukaemia<br>Leukaemia of unspecified cell type

S2. Cumulative cancer incidence (raw numbers [n], percentages [%], Crude Incidence Rate [CIR], and Standardized Incidence Rate Ratio [SIR with 95% Confidence Interval [CI]). Numbers reported are for cancer incidence, with percentages calculated from the total number of cancers not person. CIR are reported per 100,000 person-years and SIR are age- sex- standardised rate ratios.

Cancer category	Intellectual Disabilities (n=17,203)			General Population (n=566,061)			SIR (95% CI)
	n	%	CIR	n	%	CIR	
All incident cancers	816*	100.0%	464.3	43,775*	100.0%	781.8	**0.76 (0.70, 0.82)**
Digestive organs	220	26.9%	132.2	9,447	21.5%	175.6	0.95 (0.81, 1.10)
Colorectal	116	14.2%	72.5	5,090	11.6%	94.8	0.96 (0.78, 1.17)
Oesophageal	29	3.5%	19	1,055	2.4%	21.4	1.04 (0.70, 1.55)
Stomach	22	2.7%	14.9	809	1.8%	16.4	1.11 (0.71, 1.72)
Liver	14	1.7%	6.1u	833	1.9%	13.9	0.63 (0.31, 1.27)u
Pancreas	13	1.6%	8.8u	938	2.1%	19.3	0.64 (0.36, 1.15)u
Respiratory & Intrathoracic	91	11.1%	56.3	7,180	16.4%	133.8	**0.56 (0.45, 0.70)**
Lung	76	9.3%	50.8	6,104	13.9%	125.7	**0.55 (0.43, 0.70)**
Haematopoietic	75	9.2%	42.7	3,375	7.7%	58.0	0.88 (0.68, 1.14)
Hodgkin's & Non-Hodgkin's lymphoma	41	5.0%	23.7	1,519	3.5%	28.2	0.96 (0.68, 1.36)
Lymphoid leukaemia	10	1.2%	6.1u	504	1.1%	7.4	1.09 (0.54, 2.20)u
Myeloid leukaemia	5	0.6%	2.7u	432	1.0%	6.4	0.62 (0.21, 1.80)u
Leukaemia of unspecified cell type	<5	0.0%		19	0.0%	0.4u	
Metastatic cancer of unknown primary origin	57	7.0%	27.8	1,378	3.1%	21.1	**1.86 (1.34, 2.60)**
Urinary tract	56	6.8%	30.5	2,664	6.1%	46.7	0.80 (0.59, 1.09)
Kidney	34	4.2%	22.4	1,076	2.5%	22.2	1.22 (0.85, 1.76)
Bladder	13	1.6%	7.5u	1,105	2.5%	20.8	0.46 (0.25, 0.85)u
Lip, oral cavity & pharynx	21	2.6%	12.9	1,148	2.6%	21.6	0.69 (0.43, 1.11)
Central Nervous System	17	2.1%	6.1u	625	1.4%	11.1	0.60u (0.30, 1.21)u
Melanoma (skin)	17	2.1%	11.52u	1,416	3.2%	29.0	**0.47 (0.28, 0.78)u**
Non-melanoma skin cancer			*82.3*			*253.1*	***0.41 (0.34, 0.50)***
Mesothelial and soft tissue	6	0.7%	3.4u	583	1.3%	10.4	0.38 (0.15, 0.99)u
Thyroid & endocrine glands	5	0.6%	3.4u	341	0.8%	6.7	0.60 (0.24, 1.48)u
Thyroid	<5			297	0.7%	6.1	
Bone & articular cartilage	<5			81	0.2%	0.9	

* This figure excludes non-melanoma skin cancers. Figures in bold are statistically significant at 5% level.

U: unreliable age- sex- standardisation due to n<20 cases.

S3. Cancer mortality (raw numbers [n], percentages [%], Crude Mortality Rate [CMR], Standardized Mortality Ratios [SMR] with 95% Confidence Intervals [CI]), for underlying cause of death as cancer. CMR are reported per 100,000 person-years and SMR are age- sex- standardised rate ratios.

Cancer category	Intellectual Disabilities (n=17,203)			General Population (n=566,061)			SMR (95% CI)
	n	%	CMR	n	%	CMR	
Number of main-cause cancer deaths	435	100.0%	318.5	18,678	100.0%	398.8	**1.20 (1.08, 1.33)**
Digestive organs	115	26.4%	84.2	4,739	25.4%	101.2	1.21 (0.99, 1.48)
Colorectal	52	12.0%	38.1	1,741	9.3%	37.2	**1.54 (1.13, 2.08)**
Oesophageal	22	5.1%	16.1	811	4.3%	17.3	1.22 (0.76, 1.93)
Stomach	16	3.7%	11.7u	562	3.0%	12.0	1.38 (0.80, 2.35)u
Pancreas	<20		8.1u	790	4.2%	16.9	0.74 (0.39, 1.42)u
Liver	<10		5.1u	495	2.7%	10.6	0.76 (0.34, 1.72)u
Respiratory & Intrathoracic organs	66	15.2%	48.3	4,518	24.2%	96.5	**0.77 (0.59, 1.00)**
Lung	61	14.0%	44.7	4,368	23.4%	93.3	**0.75 (0.57, 0.98)**
Metastatic cancer of unknown primary origin	39	9.0%	28.6	808	4.3%	17.3	**2.64 (1.86, 3.74)**
Urinary tract	26	6.0%	19.0	875	4.7%	18.7	1.32 (0.87, 2.00)
Kidney	15	3.4%	11.0u	314	1.7%	6.7	**2.01 (1.16, 3.49)u**
Bladder	10	2.3%	7.3u	470	2.5%	10.0	1.03 (0.54, 1.99)u
Haematopoietic	26	6.0%	19.0	950	5.1%	20.3	1.25 (0.82, 1.90)
Hodgkin's & Non-Hodgkin's lymphoma	14	3.2%	10.3u	403	2.2%	8.6	1.51 (0.87, 2.60)u
Lymphoid leukaemia	<5			75	0.4%	1.6	
Myeloid leukaemia	<5			181	1.0%	3.9	
Leukaemia of unspecified cell type	<5			<5			
Lip, oral cavity & pharynx	<20		6.6u	367	2.0%	7.8	0.96 (0.48, 1.95)u
Central Nervous System	7	1.6%	5.1u	372	2.0%	7.9	0.76 (0.34, 1.70)u
Mesothelial and soft tissue	<5			286	1.5%	6.1	
Melanoma (skin)	<5			154	0.8%	3.3	
Non-melanoma skin	*<5*			*706*	*0.1%*	*15.1*	
Thyroid & other endocrine glands	<5			35	0.2%	0.8	
Thyroid	<5			23	0.1%	0.5	
Bone & articular cartilage	<5			<20		0.4u	

*The total number of cancers reported here is slightly lower than the actual number in the dataset due to mismatched sex (see section 2.1). This figure also excludes non-melanoma skin cancers. Figures in bold are statistically significant at 5% level. U: unreliable age- sex- standardisation due to n<20 cases.

S4. All-cause cancer mortality (raw numbers [n], percentages [%], Crude Mortality Rate [CMR], Standardized Mortality Ratios [SMR] with 95% Confidence Intervals [CI]), for all-cause of death as cancer. CMR are reported per 100,000 person-years and SMR are age- sex- standardised rate ratios.

Cancer category	Intellectual Disabilities (n=17,203)			General Population (n=566,061)			SMR (95% CI)
	n	%	CMR	n	%	CMR	
Number of all-cause cancer deaths	537	100.0%	393.2	22,467	100.0%	479.7	**1.29 (1.17, 1.41)**
Digestive organs	160	29.8%	117.1	6,429	28.6%	137.3	**1.26 (1.06, 1.50)**
Colorectal	70	13.0%	51.3	2,694	12.0%	57.5	**1.37 (1.06, 1.78)**
Oesophageal	25	4.7%	18.3	898	4.0%	19.2	1.20 (0.77, 1.86)
Stomach	20	3.7%	14.6u	646	2.9%	13.8	1.47u (0.92, 2.37)u
Liver	14	2.6%	10.3u	729	3.2%	15.6	1.04u (0.59, 1.85)u
Pancreas	<20		8.8u	847	3.8%	18.1	0.80u (0.43, 1.50)u
Respiratory & Intrathoracic organs	79	14.7%	57.8	5,880	26.2%	125.6	**0.74 (0.59, 0.94)**
Lung	67	2.5%	49.1	5,005	22.3%	106.9	**0.74 (0.57, 0.96)**
Unknown primary origin (metastases)	57	10.6%	41.7	1,284	5.7%	27.4	**2.53 (1.90, 3.38)**
Lymphoid, haematopoietic & related tissue	46	8.6%	33.7	1,824	8.1%	39.0	1.23 (0.90, 1.69)
Hodgkin's & Non-Hodgkin's lymphoma	24	4.5%	17.6	674	3.0%	14.4	**1.72 (1.11, 2.66)**
Lymphoid leukaemia	<10		4.4u	274	1.2%	5.9	1.30u (0.54, 3.14)u
Myeloid leukaemia	5	0.9%	3.7u	324	1.4%	6.9	0.80u (0.29, 2.20)u
Leukaemia of unspecified cell type	<5			5	0.0%	0.1u	
Urinary tract	44	8.2%	32.2	1,580	7.0%	33.7	1.37 (0.99, 1.89)
Kidney	22	4.1%	16.1	451	2.0%	9.6	**2.14 (1.36, 3.36)**
Bladder	13	2.4%	9.5u	709	3.2%	15.1	1.04u (0.58, 1.89)u
Central Nervous System	15	2.8%	11.0u	492	2.2%	10.5	1.08u (0.63, 1.87)u
Lip, oral cavity & pharynx	13	2.4%	9.5u	581	2.6%	12.4	1.04u (0.57, 1.88)u
Melanoma (skin)	6	1.1%	4.4u	280	1.2%	6.0	1.36u (0.57, 3.28)u
Non-melanoma skin	*36*	*0.2%*	*89.3*	*2,805*	*0.5%*	*257.3*	*0.50 (0.41, 0.61)*
Mesothelial and soft tissue	<5			422	1.9%	9.0	
Thyroid & other endocrine glands	<5			73	0.3%	1.6	
Thyroid	<5			39	0.2%	0.8	
Bone and articular cartilage	<5			62	0.3%	1.3	

*The total number of cancers reported here is slightly lower than the actual number in the dataset due to mismatched sex (see section 2.1 for more detail). This figure also excludes non-melanoma skin cancers. Figures in bold are statistically significant at 5% level.

S5. Female all-cause cancer mortality (raw numbers [n], percentages [%], Crude Mortality Rate [CMR], Standardized Mortality Ratios [SMR] with 95% Confidence Intervals [CI]), for all-cause of death as cancer. CMR are reported per 100,000 person-years and SMR are age- standardised rate ratios.

Cancer category	Intellectual Disabilities			General Population			SMR (95% CI)
	n	%	CMR	n	%	CMR	
Number of all-cause cancer deaths	263	100.0%	438.3	10,860	100.0 %	438.9	**1.44 (1.26, 1.64)**
Digestive organs	62	23.6%	103.3	2,857	26.3%	115.5	**1.30 (1.00, 1.70)**
Colorectal	27	10.3%	45	1,258	11.6%	50.9	1.35 (0.90, 2.03)
Oesophageal	7	2.7%	11.7u	312	2.9%	12.6	1.13 (0.52, 2.46)u
Stomach	8	3.0%	13.3u	259	2.4%	10.5	1.59 (0.75, 3.36)u
Liver	7	2.7%	11.7u	271	2.5%	11	1.64 (0.75, 3.61)u
Pancreas	<5			430	4.0%	17.4	
Respiratory & Intrathoracic organs	36	13.7%	60	2,818	25.9%	113.9	0.79 (0.56, 1.11)
Lung	30	11.4%	50	2,441	22.5%	98.7	0.77 (0.53, 1.13)
Metastatic cancers of unknown primary origin	27	10.3%	45	716	6.6%	28.9	**2.50 (1.66, 3.76)**
Haematopoietic	19	7.2%	31.7u	785	7.2%	31.7	1.32 (0.81, 2.14)u
Hodgkin's & Non-Hodgkin's lymphoma	13	4.9%	21.7u	294	2.7%	11.9	**2.31 (1.29, 4.16)u**
Lymphoid leukaemia	<5			104	1.0%	4.2	
Myeloid leukaemia	<5			159	1.5%	6.4	
Leukaemia of unspecified cell type	<5			<5			
Urinary tract	17	6.5%	28.3u	591	5.4%	23.9	**1.78 (1.08, 2.94)u**
Kidney	9	3.4%	15.0u	179	1.6%	7.2	**2.89 (1.46, 5.72)u**
Bladder	7	2.7%	11.7u	261	2..4%	10.6	1.87 (0.85, 4.12)u
Female genital organs	44	16.8%	73.3	1,089	10.0%	44	**2.22 (1.61, 3.08)**
Body of Uterus	19	7.3%	31.7u	346	3.2%	14	**3.00 (1.84, 4.90)u**
Ovary	18	6.9%	30.0u	374	3.4%	15.1	**2.75 (1.65, 4.60)u**
Vulva	<5			61	0.6%	2.5	
Cervical	<5			98	0.9%	4	
Breast	43	16.4%	71.7	1,247	11.5%	50.4	**2.07 (1.50, 2.87)**
Male genital organs							
Prostate							
Testicular							
Central Nervous System	9	3.4%	15.0u	209	1.9%	8.5	1.73 (0.87, 3.44)u
Lip, oral cavity & pharynx	5	1.9%	8.3u	174	1.6%	7	1.53 (0.62, 3.82)u
Melanoma (skin)	<5			115	1.1%	4.7	
Non-melanoma skin	*13*	*0.2%*	*81.7*	*1,110*	*0.4%*	*213.1*	***0.53 (0.39, 0.72)u***
Mesothelial and soft tissue	<5			161	1.5%	6.5	
Thyroid & other endocrine glands	<5			46	0.4%	1.9	
Thyroid	<5			27	0.2%	1.1	
Bone and articular cartilage	<5			28	0.3%	1.1	

Note: non-melanoma skin cancers were not included in the rates calculations in the denominator. Figures in bold are statistically significant at 5% level.
U: unreliable age- sex- standardisation due to n<20 cases.

S6. Male all-cause cancer mortality (raw numbers [n], percentages [%], Crude Mortality Rate [CMR], Standardized Mortality Ratios [SMR] with 95% Confidence Intervals [CI]), for all-cause of death as cancer. CMR are reported per 100,000 person-years and SMR are age- standardised rate ratios.

Cancer category	Intellectual Disabilities			General Population			SMR (95% CI)
	n	%	CMR	n	%	CMR	
Number of all-cause cancer deaths	274	100.0%	357.8	11,607	100.0 %	525.4	**1.14 (1.00, 1.30)**
Digestive organs	98	35.8%	128	3,572	30.8%	161.7	1.23 (0.99, 1.54)
Colorectal	43	15.7%	56.2	1,436	12.4%	65	1.39 (0.99, 1.94)
Oesophageal	18	6.6%	23.5u	586	5.0%	26.5	1.24 (0.73, 2.10)u
Stomach	12	4.4%	15.7u	387	3.3%	17.5	1.40 (0.76, 2.58)u
Liver	7	2.6%	9.1u	458	3.9%	20.7	0.69 (0.30, 1.56)u
Pancreas	8	2.9%	10.5u	417	3.6%	18.9	1.02 (0.47, 2.21)u
Respiratory & Intrathoracic organs	43	15.7%	56.2	3,062	26.4%	138.6	0.70 (0.51, 0.97)
Lung	37	13.5%	48.3	2,564	22.1%	116.1	0.71 (0.50, 1.02)
Metastatic cancers of unknown primary origin	30	10.9%	39.2	568	4.9%	25.7	**2.58 (1.73, 3.84)**
Haematopoietic	27	9.9%	35.3	1,039	9.0%	47	1.17 (0.77, 1.78)
Hodgkin's & Non-Hodgkin's lymphoma	11	4.0%	14.4u	380	3.3%	17.2	1.26 (0.65, 2.42)u
Lymphoid leukaemia	5	1.8%	6.5u	170	1.5%	7.7	1.88 (0.72, 4.91)u
Myeloid leukaemia	<5			165	1.4%	7.5	
Leukaemia of unspecified cell type	<5			<5			
Urinary tract	27	9.9%	35.3	989	8.5%	44.8	1.12 (0.74, 1.70)
Kidney	13	4.7%	17.0u	272	2.3%	12.3	1.64 (0.92, 2.92)u
Bladder	6	2.2%	7.8u	448	3.9%	20.3	0.56 (0.24, 1.30)u
Male genital organs	37	13.6%	48.3	1,957	16.9%	88.7	1.29 (0.91, 1.83)
Prostate	22	8.1%	28.7	1,379	11.9%	62.5	1.24 (0.80, 1.92)
Testicular	<5			15	0.1%	0.7u	
Central Nervous System	6	2.2%	7.8u	283	2.4%	12.8	0.61 (0.26, 1.46)u
Lip, oral cavity & pharynx	8	2.9%	10.5u	407	3.5%	18.4	0.82 (0.37, 1.81)u
Melanoma (skin)	<5			165	1.4%	7.5	
Non-melanoma skin	*23*	*0.2%*	*95.3*	*1,695*	*0.6%*	*306.8*	***0.47 (0.37, 0.61)***
Mesothelial and soft tissue	<5			261	2.2%	11.8	
Thyroid & other endocrine glands	<5			27	0.2%	1.2	
Thyroid	<5			12	0.1%	0.5u	
Bone and articular cartilage	<5			34	0.3%	1.5	

Note: non-melanoma skin cancers were not included in the rates calculations in the denominator. Figures in bold are statistically significant at 5% level.
U: unreliable age- sex- standardisation due to n<20 cases.
