# Supplementary material for: Population-based cancer incidence and mortality rates and ratios among adults with intellectual disabilities in Scotland: a retrospective cohort study with record linkage
Source: BMJ Open. 2024 Aug 12;14(8):e084421. doi: 10.1136/bmjopen-2024-084421 (PMC11331995; doi:10.1136/bmjopen-2024-084421)
Supplement: Supplementary file 2 [file bmjopen-2024-084421.reviewer_comments.pdf]

PEER REVIEW HISTORY

BMJ Open publishes all reviews undertaken for accepted manuscripts. Reviewers are asked to complete a checklist review form (**http://bmjopen.bmj.com/site/about/resources/checklist.pdf**) and are provided with free text boxes to elaborate on their assessment. These free text comments are reproduced below.

ARTICLE DETAILS

**TITLE (PROVISIONAL)**	Population-based cancer incidence and mortality rates and ratios among adults with intellectual disabilities in Scotland: A retrospective cohort study with record linkage
**AUTHORS**	Ward, Laura; Cooper, Sally-Ann; Sosenko, Filip; Morrison, David; Fleming, Michael; McCowan, Colin; Robb, Katie; Hanna, Catherine; Hughes-McCormack, Laura; Dunn, Kirsty; Conway, D; Henderson, Angela; Smith, Gill; Truesdale, Maria; Cairns, Deborah

VERSION 1 – REVIEW

**REVIEWER**	Cacho-Díaz, Bernardo<br>Instituto Nacional de Cancerología, Neuro-oncology
**REVIEW RETURNED**	01-Feb-2024

**GENERAL COMMENTS**	This is a relevant and well written study.

**REVIEWER**	Satgé, Daniel<br>Institut Universitaire de Recherche Clinique
**REVIEW RETURNED**	21-Feb-2024

**GENERAL COMMENTS**	This is a very interesting work which answers the questions on differences between<br>cancer incidence and cancer mortality of persons with intellectual disability (ID). It is<br>based on high quality sources in a country which has a good knowledge on ID.<br>The review of the literature on the subject is well conducted and well analyzed,<br>indicating a good understanding of this subject. It is well written (as far as a non English native person can evaluate).<br>Data are strong and make this article a major contribution in this field.<br>The discussion is well conducted, the conclusion provides useful messages.<br>Tables are well built, figures clearly illustrate the data.<br>Minor points<br>Page 16 lines 23 and 24 the sequence ''ovarian (1.59)'' appears twice, the second is to<br>be deleted.<br>Page 17 line 4 delete the words ''benign cancer'' a cancer is by definition never<br>benign. Rather use ''benign neoplasm'' or ''benign tumor''.

	Apologizes for this answer out of the website.<br>Do not hesitate to ask if you have any question, or a template to be filled<br>and easy to find.

**REVIEWER**	Srasuebkul, Preeyaporn<br>UNSW Australia, Department of Developmental Disability Neuropsychiatry
**REVIEW RETURNED**	21-Feb-2024

**GENERAL COMMENTS**	The "Population-based cancer incidence and mortality rates and ratios among adults with intellectual disability in Scotland" aimed to provide contemporary data on cancer mortality rates within the context of incidence in the population with intellectual disabilities.<br><br>The authors compared the rates using an the indirect standardised method, which is appropriate for their research question.<br><br>I have a few minor comments for the authors to consider<br>1. The main aim of this paper is to compare the rate of cancer in people with and without intellectual disability; some cancers were too few in people with intellectual disability to reliably calculate the ratio. I think the authors should not present these cancers in the main tables; it distracts readers. The full table should be given in the appendix.<br>2. When we discuss causes of death, there is standard terminology; the terminology for what authors refer to as 'main cause' is 'underlying cause'. I suggest the authors use the standard term, as everyone easily understands it.

VERSION 1 – AUTHOR RESPONSE

Response to Reviewers

Thank you kindly to Dr Cacho Díaz, Dr Satgé, and Dr Srasuebkul for your time, expertise, and feedback on the manuscript. The revisions have strengthened the paper and we as a research team, appreciate your efforts.

1. Minor points Page 16 lines 23 and 24 the sequence ‘’ovarian (1.59)’’ appears twice, the second is to be deleted.

The manuscript has been edited accordingly.

2. Page 17 line 4 delete the words ‘’benign cancer’’ a cancer is by definition never benign. Rather use ‘’benign neoplasm’’ or ‘’benign tumor’’.

The manuscript has been edited accordingly.

3. The main aim of this paper is to compare the rate of cancer in people with and without intellectual disability; some cancers were too few in people with intellectual disability to reliably calculate the ratio. I think the authors should not present these cancers in the main tables; it distracts readers. The full table should be given in the appendix.

There is always a difficulty with balancing statistical disclosure control mechanisms with utility. Whilst we appreciate that being able to focus on ‘positive’ larger results may be beneficial, we think there is more value in presenting a complete overview of neoplasms with the full data. The ONS states “deaths and rates based on 10 to 19 deaths are marked with a “u” to warn users that their reliability is low” [1]. However, other academic papers fail to follow this best practice simply reporting the rates

(e.g. Sullivan et al., 2003 [2]). We believe presenting the current tables is more informative and shows where rates are small, and importantly we make no claims that are not justified by the data.

4. When we discuss causes of death, there is standard terminology; the terminology for what authors refer to as 'main cause' is 'underlying cause'. I suggest the authors use the standard term, as everyone easily understands it.

We have aligned with this phrasing and changed 'main-cause' to 'underlying cause'

References

[1] Office for National Statistics (2022) Avoidable mortality in the UK Quality and Methodology Information (https://www.ons.gov.uk/peoplepopulationandcommunity/healthandsocialcare/causesofdeath/methodologies/avoidablemortalityinenglandandwalesqmi)

[2] Sullivan, S. G., Glasson, E.J., Hussain, R., Petterson, B.A., Slack-Smith, L.M., Mongomery, P.D., and Bittles, A.H. (2003) Breast cancer and the uptake of mammography screening services by women with intellectual disabilities. 10.1016/S0091-7435(03)00177-4

VERSION 2 – REVIEW

**REVIEWER**	Satgé, Daniel<br>Institut Universitaire de Recherche Clinique
**REVIEW RETURNED**	13-Jun-2024

**GENERAL COMMENTS**	This is a very interesting result, important for future works on cancer in persons with intellectual disability.<br>I am not sure that testicular cancer has not been suspected to be increased in meb with ID. This dores not modify the quality of your article. See Annie J. Sasco, Roland Ah-Song, Motoi Nishi, Stéphane Culine, Marie-Odile Réthoré, et al.. Testicular cancer and intellectual disability. International journal on disability and human development, 2008, 7 (4), pp.399-405. inserm-00289912

**REVIEWER**	Srasuebkul, Preeyaporn<br>UNSW Australia, Department of Developmental Disability Neuropsychiatry
**REVIEW RETURNED**	07-Jun-2024

**GENERAL COMMENTS**	I have no further comments, as the authors satisfactorily addressed all concerns.

VERSION 2 – AUTHOR RESPONSE

Response to Reviewers

Thank you Dr Satge for raising this review to our attention, we have referenced this in the discussion and removed the text alluding to the novelty of the testicular cancer finding.
